# Dual roles and potential applications of exosomes in HCV infections

**DOI:** 10.3389/fmicb.2022.1044832

**Published:** 2022-12-12

**Authors:** Yiqian Yin, Yuxue Zhao, Qiaoqiao Chen, Yiwen Chen, Lingxiang Mao

**Affiliations:** Department of Laboratory Medicine, Affiliated Kunshan Hospital of Jiangsu University, Suzhou, Jiangsu, China

**Keywords:** exosomes, hepatitis C virus, viral RNA, non-coding RNAs, diagnosis, treatment

## Abstract

The hepatitis C virus (HCV) causes severe liver diseases, including hepatitis, liver cirrhosis, and hepatocellular carcinoma, which have high morbidity and mortality. Antibody targeting receptor-mediated HCV infections have limited therapeutic benefits, suggesting that the transmission of HCV infections is possibly mediated *via* receptor-independent mechanisms. Exosomes are membrane-enclosed vesicles with a diameter of 30–200 nm, which originate from the fusion of endosomal multivesicular bodies with the plasma membrane. Accumulating evidence suggests that exosomes have a pivotal role in HCV infections. Exosomes can transfer viral and cellular bioactive substances, including nucleic acids and proteins, to uninfected cells, thus spreading the infection by masking these materials from immunological recognition. In addition, exosomes originating from some cells can deliver antiviral molecules or prompt the immune response to inhibit HCV infection. Exosomes can be used for the diagnosis of HCV-related diseases, and are being presently evaluated as therapeutic tools for anti-HCV drug delivery. This review summarizes the current knowledge on the dual roles and potential clinical applications of exosomes in HCV infections.

## Introduction

The hepatitis C virus (HCV) is a positive-stranded, enveloped RNA virus belonging to the *Flaviviridae* family. HCV is a major cause of chronic hepatitis, cirrhosis, and hepatocellular carcinoma (HCC), and infects approximately 130–170 million people worldwide (Kim et al., 2016). Owing to its high genetic diversity, seven genotypes of HCV have been recognized (1–7), of which genotypes 1, 2, and 3 are more prevalent than the others (Smith et al., 2014). The 9.6-kb RNA genome of HCV is translated into a polyprotein that is cleaved into 10 mature products, including three structural proteins (core protein and glycoproteins E1 and E2) and seven nonstructural proteins (p7, NS2, NS3, NS4A, NS4B, NS5A, and NS5B) (Lohmann et al., 1999; Lindenbach and Rice, 2013).

The early endosomes formed by the fusion of endocytic vesicles are converted into multivesicular bodies (MVBs) that pack with intraluminal vesicles (ILVs). The MVBs subsequently target the cell membrane to release the ILVs into the extracellular domain, which are the exosomes. Exosomes are double-layered nano-sized vesicles that have received considerable attention as an important tool for intercellular communication, owing to their ability to transfer various biological substances to target cells (Kowal et al., 2014). Exosomes are found in all biological fluids and are secreted by all cell types. These features render them attractive as “minimally invasive liquid biopsies” with the potential for longitudinal sampling for studying disease progression (Soda et al., 2019).

After removing cells by low-speed centrifugation, exosomes can be isolated by filtration (FC) or polymer-based precipitation (P) methods [such as using polyethylene glycol (PEG)], differential centrifugation (dUC) with increasing *g* force/time, immunoprecipitation (IP) with specific antibodies, asymmetric flow field-flow fractionation, or density gradient separation. These conventional methods can lead to contamination by other extracellular vesicles (EVs). Researchers have therefore proposed a simple and label-free approach based on surface plasmon resonance for isolating and quantifying tumor-relevant exosomes from bulk exosomes (Sina et al., 2016). Exosomal microRNAs (miRNAs) were previously analyzed by quantitative real-time reverse transcription-quantitative polymerase chain reaction (RT-qPCR) or microarrays, and exosomal proteins were analyzed by western blotting, enzyme-linked immunosorbent assay (ELISA), and mass spectrometry. However, these methods are time-consuming and labor-intensive, and therefore impractical for clinical application.

Several novel approaches have been employed to address the aforementioned concerns. Boriachek et al. (2018) invented an amplification-free electrochemical method for the rapid detection of exosomal miRNA-21 from complex serum samples. They used magnetic beads with complementary probes for capturing the target miRNA, and the level of adsorbed miRNA was subsequently monitored using an electrochemical approach in the presence of a redox system. In earlier studies, a microfluidic platform technology based on alternating current electrohydrodynamic induced “nanoshearing” was employed for capturing and detecting exosomal targets from cell cultures and clinical samples. However, the structure and performance of the device required optimization for clinical applications (Vaidyanathan et al., 2014). Unlike the electronic method, a stripping voltametric immunoassay using quantum dots as signal amplifiers was used in a study to detect low concentrations of exosomes in small clinically relevant samples of colon cancer (Boriachek et al., 2017). Flow cytometry is used widely for the high-throughput quantitative and qualitative analyses of exosomal proteins. This method is based on the detection of scatter and fluorescent light for individual exosomes. Flow cytometry can be modified for sorting exosomes and microvesicles according to the exosomal transmembrane proteins, including CD81, CD63, and CD9. However, flow cytometry might not allow the detection of weak signals as it largely depends on the abundance of exosomal antigens (Pospichalova et al., 2015; Wu et al., 2015). A previous study reported a simple, highly sensitive, portable exosome detection system for the diagnosis of breast cancer, based on the identification of the exosomal tumor markers, human epidermal growth factor receptor 2 and epithelial cell adhesion molecules. The method is based on the detection of surface-enhanced Raman scattering using surface-enhanced Raman scattering gold nanorods (Kwizera et al., 2018). Additionally, several exosome detection methods based on superparamagnetic nanoarchitectures were introduced in a review by Masud et al. (2019). The new methods for the detection of exosomal contents are summarized in Table 1.

**Table 1:** Summary of new technologies for the detection of exosomal contents.

Detection method	Detection target	Advantages	Limitations	Limit of detection	Detection of clinical diseases in published studies	References
Amplification-free electrochemical method	Exosomal miRNAs	Simple, inexpensive, and rapid	_	1 pM exosomal miRNA	Colorectal adenocarcinoma	Boriachek et al. (2018)
Microfluidic platform technology	Exosomal surface proteins	Rapid, multiplexed, and highly specific	Not suitable for small samples	2.76 × 10^3^ exosomes/μL	Breast cancer	Vaidyanathan et al. (2014)
Stripping voltametric immunoassay	Exosomal proteins	Highly specific and sensitive, applicable to small samples	_	100 exosomes/μL	Colon cancer	Boriachek et al. (2017)
Flow cytometry	Exosomal proteins, separating exosomes from other microvesicles	Simple	Cannot detect weak light scattered from exosomes	_	Ovarian cancer	Pospichalova et al. (2015), Wu et al. (2015)
Surface-enhanced Raman spectroscopy	Exosomal surface proteins	Simple, rapid, and highly sensitive; used widely in clinical medicine	Initially requires exosomes for isolation	2 × 10^3^ exosomes/mL	HER2-positive breast cancer	Kwizera et al. (2018)

This review summarizes the studies on the functions and mechanism of action of exosomes in HCV infections. The potential applications of exosomes for the diagnosis and treatment of HCV-related diseases are additionally discussed.

## Dual roles of exosomes in HCV infection

### Exosomes can facilitate HCV infections by transferring various biological activators

#### Exosomes transfer viral components

Ramakrishnaiah et al. were the first group to demonstrate that exosome-derived hepatoma cells infected with HCV contain complete viral genomes, viral proteins, and particles. These substances were transported to uninfected hepatoma cells to establish a productive infection in the presence of neutralizing antibodies (Ramakrishnaiah et al., 2013). Exosomes provide a protective cover that supports persistent HCV infections by enabling the viral particles to escape the innate antiviral response (Aydin et al., 2021). Liu and colleagues detected HCV virions in exosomes by transmission electron microscopy, and identified the presence of HCV RNA, E2 proteins, and core proteins in the exosomes by real-time RT-qPCR and western blotting. The findings revealed that HCV RNA was predominantly present in the exosomes isolated from the plasma of patients infected with HCV. Although the infectivity of the exosome-associated HCV particles was lower than that of the free HCV particles, the exosome-associated HCV particles were found to be more infectious and resistant to anti-HCV antibodies than the free HCV particles (Liu et al., 2014).

Bukong et al. demonstrated that exosomes released from the infected hepatocytes and sera of patients with chronic HCV infection contained viral RNA in complex with miRNA-122, Ago2, and HSP90. The findings revealed that exosomes could mediate the transmission of HCV to uninfected hepatocytes independent of the CD81 viral receptor, scavenger receptor type B1, E2, or apolipoprotein E. These Ago2-miRNA-122-HSP90 complexes possibly stabilized the exosomal HCV RNA-replication complex, which resulted in the higher infectivity of the exosome-associated HCV particles compared to that of the free HCV particles. The study also demonstrated the presence of replication-competent HCV RNA in the exosomes isolated from the sera of HCV-infected treatment-non-responders and several treatment-naïve patients, and these findings can be used to explore the treatment resistance of infected patients by interferon (IFN) therapy or ribavirin (Bukong et al., 2014). Exosomes can be infected with free HCV particles owing to the similarities in the size, density, and sedimentation characteristics of exosomes and infectious virions. HCV subgenomic replicon (SGR) cells that lack viral structural proteins and cannot produce infectious virions have been used to study the exosome-mediated transfer of HCV RNA between hepatocytes. It has been demonstrated that exosomes from SGR cells can transfer replication-competent HCV RNA to uninfected Huh7 cells independent of infectious virions. Contrary to other reports (Ramakrishnaiah et al., 2013; Bukong et al., 2014; Liu et al., 2014), Longatti and coworkers demonstrated that the concentration of exosomes secreted from SGR cells is not sufficiently high for establishing infection unless it occurs *via* cell–cell contact. This pathway of viral dissemination and immune escape is inefficient as only 0.1% of the exosomes isolated from the culture media of SGR cells were found to contain HCV RNA (Longatti et al., 2015).

To date, further studies are necessary for completely elucidating whether defective interfering (DI) viruses maintains chronic infection or viral clearance in the host. HCV in-frame deletion mutants (IFDMs), which are associated with chronic HCV infection, have an increased viral replication rate and promote viral release in the presence of full-length RNA. Karamichali et al. were the first to detect IFDMs in exosomes derived from the sera of chronically infected patients with IFDMs belonging to genotype 3a, and reported that IFDMs can exploit exosomes for spreading HCV infection. Inhibition of the release of exosomes containing IFDMs increases the intracellular accumulation of core proteins and viral replication, which can lead to the establishment of a persistent infection in the host cells (Karamichali et al., 2018). Exosomes not only serve as carriers between hepatocytes but also act as important modulators between hepatocytes and non-permissive “bystander” cells. It has been reported that exosomes derived from the plasma of HCV-infected patients and the supernatants of HCV-infected hepatocytes can transmit HCV RNA to myeloid-derived suppressor cells (MDSCs) and promote the expansion of MDSCs by downregulating the expression of miRNA-124. The expansion of MDSCs can subsequently facilitate the differentiation of T follicular regulatory (Tfr) cells, increase the production of interleukin (IL)-10, inhibit the function of T follicular helper (Tfh) cells, and suppress the secretion of IFN-γ, which can lead to persistent viral infections. A previous study outlined a novel mechanism which described that exosomes dysregulate the immune response during HCV infections (Wang et al., 2018). HCV-associated exosomes reduce the expression of miRNA-124 by in myeloid cells by two mechanisms which are described hereafter. First, exosomes can upregulate the expression of HOXA transcript antisense RNA myeloid-specific 1 (HOTAIRM1) and its target, HOXA1, in MDSCs and subsequently stimulate the production of immunosuppressive factors by controlling the expression of miRNA-124 (Thakuri et al., 2020a). Second, Thakuri et al. suggested that the increased expression of a long non-coding RNA-RUNX1 overlapping RNA (RUNXOR) and its downstream runt-related transcription factor 1 induced by the HCV-associated exosomes (HCV-exo) to inhibit the expression of miRNA-124 *via* the signal transducer and activator of transcription 3 (STAT3) pathway, which in turn promotes the immunosuppressive functions of MDSCs (Thakuri et al., 2020b). Because signaling networks are highly complex, the possible mechanisms underlying the mechanism related to HCV-exo should be explored in future studies for elucidating the occurrence of persistent HCV infections.

Galectin-9 (gal-9) is a natural ligand of the T cell immunoglobulin domain and mucin domain protein 3. The expression of gal-9 increases significantly in the sera and liver tissues of patients with chronic HCV infection. The production of gal-9 in monocytes and macrophages (MΦs) following induction by IFN-γ can expand the number of suppressive regulatory T cells and accelerate the apoptosis of HCV-specific cytotoxic T lymphocytes (CTLs), which in turn helps in maintaining chronic HCV infection (Mengshol et al., 2010). It has been demonstrated that exosomes from SGR and infected cells containing HCV RNA can stimulate the production of gal-9 in monocytes, and that the differentiation of monocytes also increases the levels of gal-9. Compared to normal controls, patients with HCV have higher levels of gal-9 in classic and nonclassic monocytes, of which the highest levels of gal-9 have been observed in nonclassic monocytes. By employing *in vitro* experiments, a previous study demonstrated that HCV RNA stimulates the production of gal-9 in nonclassic monocytes partly *via* exosomes, which in turn contributes to chronic HCV infection (Harwood et al., 2016). Some patients with acute HCV infection progress toward developing chronic infection, leading to liver fibrosis, which is generally regulated by the activation of hepatic stellate cells (HSCs). Saha et al. demonstrated that free HCV particles and HCV-infected hepatoma cells can both induce the differentiation of monocytes to MΦs that produce mixed M1/M2 cytokines and express M2 surface markers. Transforming growth factor beta (TGF-β) produced during the chronic presence of HCV particles programs monocytes and M2-MΦ-activated HSCs, which in turn promotes liver fibrosis during chronic infection (Saha et al., 2016). The authors subsequently demonstrated that exosomes isolated from the sera or hepatocytes of patients infected with HCV contain single-strand (ss) RNA. These exosomes can transfer HCV particles to naïve hepatocytes and induce monocytic differentiation, MΦ polarization, and generation of circulating fibrocytes by activating TLR7/8 during HCV infections. These findings suggested that exosomes containing ssRNA from infected hepatocytes can promote liver fibrosis by inducing the differentiation of monocytes (Saha et al., 2017). Table 2 summarizes the recent studies on exosomes carrying viral components that trigger further infection.

**Table 2:** Exosomes promote HCV infection by transporting viral components.

Donor cells	Materials carried and transported	Receptor cells	Mechanism of action	Pathogenic role of exosomes	Clinical relevance of exosomes in HCV infections	References
HCV-infected Huh7.5 cells	Full-lengthRNA, proteins, and viral particles	Naïve hepatocytes	Exosomes can transmit viral components to uninfected hepatocytes and partly resist neutralizing antibodies	_	_	Ramakrishnaiah et al. (2013)
Infected Huh7.5 cells and sera of patients with chronic HCV infection	HCV-RNA	Naïve hepatocytes	Exosomes containing viral RNA in complex with Ago, miRNA-122, or HSP90 can transfer infection to uninfected hepatocytes independent of the viral receptors, CD81, SB-RI, or APOE	HCV can be transmitted by exosomes. Treatment resistance in HCV could be attributed to the exosomes containing replication-competent HCV RNA	Exosomes from the sera of HCV-infected treatment non-responders and treatment-naïve patients contain replication-competent HCV RNA. HCV RNA cannot be detected in the exosomes derived from the sera of HCV-treatment responders	Bukong et al. (2014)
Supernatant of HCV SGR cells	Replication-competent HCV-RNA	Huh7 cells	Exosomes derived from HCV SGR cells transmits HCV RNA to Huh7 cells	_	_	Longatti et al. (2015)
Sera of patients infected with HCV IFDMs of genotype 3a	HCV IFDMs	_	IFDMs can increase viral replication and release *via* an exosomal pathway	Exosomes can carry and spread IFDMs, escape from the immune system, and possibly lead to persistent infection	Exosomes derived from serum samples of patients with chronic infection of HCV IFDM of genotype 3a	Karamichali et al. (2018)
Supernatants of HCV-infected Huh7 cells, and plasma of HCV-infected patients	HCV-RNA	MDSCs	Exosomes carrying HCV RNA can enhance the expansion of MDSCs by inhibiting the expression of miR-124, which in turn regulates T-cell differentiation	Exosomes can provide an immune evasion pathway by causing immune dysregulation in patients with chronic HCV infections	HCV RNA was detected in exosomes derived from the plasma of HCV-infected patients, but not in those derived from healthy individuals or patients treated with DAAs having a sustained virological response	Thakuri et al. (2020b)
HCV-infected cells and HCV genotype- 2 SGR cells	HCV-RNA	Monocytes	Exosomes from HCV- infected cells and SGR cells stimulates the production of gal-9 in monocytes. Gal-9 expands regulatory T cells and promotes the apoptosis of CTLs, which maintain chronic infection	Increased expression of gal-9 in monocytes is partly induced by exosomes contributing to chronic HCV infection	Expression of gal-9 was highest in nonclassic monocytes of patients with chronic infection	Harwood et al. (2016)
Huh7.5/JFH-1 cells and sera of patients with chronic HCV infection	HCV-ss RNA	Monocytes	ssRNAs packaged in exosomes induce the differentiation of monocytes and polarization of MΦ *via* the activation of TLR7/8	Exosomes derived from infected hepatocytes trigger monocytes to differentiate to M2 MΦs which secrete M1/M2 cytokines *via* TLR7/8	Exosomes isolated from the sera of HCV-infected patients contain HCV ssRNA. The number of M2 markers expressing monocytes in the circulation of HCV-infected patients and percentage of fibrocytes expressing collagen is increased in HCV-infected patients with high TLR7/8 expression	Saha et al. (2017)

#### Exosomes as carriers of non-coding RNAs

Exosomes also participate in intercellular communication between infected hepatocytes and HSCs by transporting certain miRNAs, which promotes the development of liver fibrosis. Some studies have demonstrated that miRNA-19a from HCV-infected hepatocytes is packaged into exosomes for targeting STAT3 in HSCs. This promotes the production of fibrogenic markers *via* activation of the STAT3-mediated TGF-β signaling pathway, which ultimately induces liver fibrosis. Using *in vivo* experiments, some studies have also demonstrated that exosomes isolated from the sera of HCV-infected patients suffering from liver fibrosis are more enriched in miRNA-19a than those obtained from healthy volunteers and non-HCV-related patients with liver disease (Devhare et al., 2017). Similarly, Kim et al. observed that the expression of miRNA-192 is upregulated in HCV-infected hepatocytes, and miRNA-192 can be delivered to HSCs *via* exosomes where it stimulates the transdifferentiation of HSCs into myofibroblasts *via* the activation of TGF-β1 (Kim et al., 2019). Further studies are necessary for understanding the pathogenesis of liver fibrosis in chronic HCV infections, and for determining whether exosomal miRNA-192 can serve as a potential therapeutic target for HCV in future.

The occurrence of mixed cryoglobulinemia (MC), an HCV-associated extrahepatic manifestation, is related to the exosomal miRNAs of HCV-infected hepatocytes. The miR-122/let-7b/miR-206 in HCV -infected hepatocyte-derived exosomes and HCV patient-derived exosomes can both upregulate B-cell activating factors (BAFF) through acting on TLR7 in macrophages. Then, the exo-miR-122-induced BAFF stimulate B cell activation and thus produce anti-HCV antibodies, which is associated with HCV-MC development (Liao et al., 2021).

lncRNAs are transcripts that do not encode proteins but can interact with other molecules, including proteins, DNA, and RNA (Ferrasi et al., 2020). The differential expression of lncRNAs has been identified in HCV infections. For instance, it has been reported that the expression of the lncRNAs, nuclear-enriched abundant transcript 1 and taurine upregulated gene 1, is reduced in the sera of patients with HCV and HCV-infected patients with HCC (Mohyeldeen et al., 2020). Various lncRNAs with upregulated expression in HCV patients have been described as proviral factors that are conducive to the spread of HCV infections. The expression of certain lncRNAs, including the lncRNA insulin-like growth factor 2 antisense (Xiong et al., 2015), lncRNA HOTAIR (Li et al., 2016), and lncRNA-HULC (Kitabayashi et al., 2020), is upregulated following the viral invasion of host cells. The upregulated expression of these lncRNAs contributes to the spread of viral infections by assisting viral replication and release. Also, lncRNAs-activated by TGF-β (lncRNA-ATB) can activate HSCs to accelerate liver fibrosis during HCV infections by regulating the miRNA-200a/β-catenin regulatory axis (Fu et al., 2017). Further studies are necessary for determining whether these lncRNAs are loaded into exosomes for expanding viral infections. Zhang et al. demonstrated that the expression of lncRNA-HEIH is increased in the exosomes and sera of HCV-infected patients with HCC (Zhang et al., 2018); however, the cohort considered in the study was small. Using exosomes isolated from the tissues and sera of HCV-infected patients with HCC following curative HCC resection, Wang et al. suggested that the expression of lnc-antagonizing non-protein coding RNA (lnc-DANCR) in exosomes is positively correlated to the recurrence of HCV-HCC (Wang et al., 2021). These exosomal lncRNAs serve as biomarkers of HCV-related disease, but the specific roles of these exosomal lncRNAs remain to be elucidated.

#### Exosomes function as carriers of proteins

The majority of HCV patients become persistent infection which probably stems from multiple strategies to evade the immune system. Syntenin is a major intracellular adaptor protein that is involved in exosomal biogenesis and cargo loading. It has been reported that HCV infected Huh7.5.1 cells and primary human hepatocytes (PHHs) that are rich in syntenin can secrete more E2-coated exosomes. These E2-decorated exosomes could assist the spread of HCV infections by escaping neutralization by the E2-specific antibody or chronic-phase patient serum (Deng et al., 2019). TGF-β plays an important role in the regulation of regulatory T (Treg) cells. T follicular regulatory (Tfr) cells, a subset of Tregs, could regulate the germinal center (GC) responses by limiting T follicular helper (Tfh) cells and B cells. Cobb et al. reported that exosomes derived from HCV-infected hepatocytes with a significantly higher expression of TGF-β on the surface, contributes to the expansion of Tfr cells by transferring TGF-β, which leads to the suppression of Tfh cells. Considering the increased numbers of Tfr cells in the livers of chronically-infected HCV patients, this study described a novel pathway in which TGF-β-containing exosomes derived from HCV-infected hepatocytes disrupts the function of CD4+ T cells to promote viral persistence in chronic infection (Cobb et al., 2018). Table 3 summarizes the roles of exosomal loading proteins and non-coding RNAs in facilitating HCV infections.

**Table 3:** Exosomes promote infection by carrying non-viral components.

Donor cells	Non-viral materials carried	Receptor cells	Mechanism of action	Pathogenic role of exosomes	Clinical relevance of exosomes in HCV infection	References
HCV-infected hepatocytes	miRNA-19a	HSCs	Exosomes transmit miRNA-19a to target suppressor of cytokine signaling 3 (SOCS3) proteins for activating HSCs through the STAT3 pathway	Exosomes can transmit miRNA-19a to HSCs leading to liver fibrosis	Exosomes from the sera of patients with chronic HCV infection and fibrosis has higher levels of miRNA-19a than those of healthy volunteers and non-HCV-related patients with liver-fibrosis	Devhare et al. (2017)
JFH-1 stable cells	miRNA-192	HSCs	Exosomes containing miRNA-192 stimulates the transformation of HSCs into myofibroblasts by upregulating the expression of TGF-β1	_	_	Kim et al. (2019)
HCV-infected hepatocytes	miRNA-122, miRNA-let-7b, miRNA-206		Exosomal miRNA-122/let-7b/206 can upregulate B-cell activating factors by stimulating TLR7 in macrophages	Exosomal miRNA-122/let-7b/206 promotes the development of mixed cryoglobulinemia	-	Liao et al. (2021)
Infected Huh7.5.1 cells and PHHs	E2 protein	_	E2-coated exosomes can reduce viral susceptibility for the neutralization of E2-specific antibody and the sera of patients in the chronic phase of infection	E2-coated exosomes from sera may weaken the efficacy of neutralizing antibody and aid viral escape	E2-coated exosomes have been detected in the sera of patients in the chronic phase of infection	Deng et al. (2019)
HCV-infected hepatocytes, Huh7.5.1, and PHHs	TGF-β	CD4 + T cells	Exosomes containing TGF-β contributes to the expansion of Tfr cells, which suppresses Tfh cells and induces viral persistence	Accumulation of Tfr cells in the liver tissues of HCV patients is induced by exosomal TGF-β and may contribute to viral persistence	Substantial population of Tfr cells were observed in the liver tissues of patients with chronic HCV infection, while no Tfr cells observed in nonviral hepatitis or healthy controls	Cobb et al. (2018)

### Exosomes can limit HCV infections

The innate immune response is the first line of defense against viral infections. Viral nucleic acids or proteins are recognized as antigens by the pattern-recognition receptors of immune cells. Immune cells, including MΦs, plasmacytoid dendritic cells (pDCs), natural killer (NK) cells, B cells, and T cells, produce a series of reactions to resist infection following the entry of HCV particles in the human body. For instance, HCV-infected hepatocytes and other immune cells produce type-I IFNs (IFN-α/β), type-III IFNs (IFN-λ), and type-II IFNs (IFN-c) for inhibiting viral infection (Dustin, 2017). Previous studies have demonstrated that hepatocytes preferentially express type-III IFNs following the occurrence of HCV infections (Thomas et al., 2012), which increases intrahepatic IFN-stimulated gene expression for resisting infections (Sarasin-Filipowicz et al., 2008; Charles and Dustin, 2011). MΦs also play a crucial role in hepatic antiviral immunity that protects hepatocytes from HCV infections. Exosomes isolated from the supernatants of TLR-3-activated MΦs have been shown to deliver anti-HCV factors and members of the miRNA-29 family to infected hepatocytes for inhibiting HCV infections. These findings revealed a novel mechanism in which MΦs confer immune protection to infected hepatocytes *via* exosomes (Zhou et al., 2016). Cai et al. demonstrated that following stimulation by IFNs, MΦs initially secrete certain cytokines that mediate short-acting antiviral activities, and subsequently secrete EVs that have long-lasting inhibitory effects on HCV replication. The study revealed that the antiviral effects of MΦ-derived EVs are mediated *via* marked alterations in the lncRNAs of EVs; however, details of the molecular mechanism underlying the roles of EVs require further investigation (Cai et al., 2018). Exosomes originating from HCV-infected Huh7.5.1 cells and HCV SGR cells can transfer HCV RNA to uninfected nonpermissive pDCs which prompt the production of IFN by the pDCs in response to viral infections. The innate immune response induced by intra-exosomal HCV RNA in neighboring uninfected pDCs, ESCRT-proteins, and ANXA2 is necessary for the transfer of exosomes containing HCV RNA (Dreux et al., 2012).

Human liver sinusoidal endothelial cells (HLSECs) constitute approximately 50% of the non-parenchymal cells in the liver. HLSECs have several receptors that enable the attachment and entry of HCV particles. Following the entry of HCV particles into HLSECs, the HCV RNA promotes IFN production which subsequently upregulates the production of IFN-λ3 and viperin. This leads to the inhibition of HCV replication in infected hepatocytes and induces the expression of antiviral genes in uninfected hepatocytes. Giugliano et al. observed that exosomes derived from the supernatant of HLSECs being stimulated with HCV-induces autocrine interferon signaling can suppress HCV replication in neighboring infected hepatocytes in a dose-dependent manner (Giugliano et al., 2015). Qian et al. reported that umbilical mesenchymal stem cells (uMSCs) secrete large amounts of exosomes that can repress HCV replication by transporting four types of miRNAs, including Let-7f, miRNA-145, miRNA-199a, and miRNA-221, to infected cells. These miRNAs play a crucial role in the antiviral effect by mediating the binding of RNA-induced silencing complexes to HCV RNA (Qian et al., 2016a). MSC-derived exosomes can emerge as therapeutic tools for HCV infections in future owing to their availability and low toxicity. Table 4 demonstrates that exosomes originating from immune cells and other cells limit infection *via* different biological pathways. These antiviral pathways also regulate viral and cellular lncRNAs, of which the latter also affect the replication and release of HCV (Unfried and Fortes, 2020). These viral and cellular lncRNAs include lncRNA-GAS5 (Qian et al., 2016b) and lncRNA-IFN-α-inducible protein 6 (IFI6) (Liu et al., 2019), among others. However, further studies are necessary for elucidating whether these lncRNAs are present in exosomes secreted from immune cells, HLSECs, and uMSCs.

**Table 4:** Exosomes limit HCV infection.

Type of donor cells	Donor cells	Material carried	Receptor cells	Mechanism	Clinical relevance of exosomes in HCV infection	References
Immune cells	Macrophages	Member of miRNA-29 family	HCV-infected hepatocytes	Macrophages can confer anti-HCV activity to infected hepatocytes by transmitting members of the anti-HCV miRNA-29 family *via* exosomes	_	Zhou et al. (2016)
	IFN-pulsed macrophages		Huh-7.5 cells harboring subgenomic HCV replicons	EVs of IFN-pulsed macrophages or from the sera of patients with acute/chronic HCV infection have long-lasting inhibitory effect on HCV replication	Exosomes from HCV- infected patients treated with pegylated IFN-α and ribavirin can inhibit HCV replication, while exosomes/microvesicles from healthy controls or patients with acute HCV infections before antiviral therapy lack inhibitory effect	Cai et al. (2018)
Other cells	HCV-infected cells and HCV SGR cells	HCV-RNA	pDCs	Exosomes from infected cells and SGR cells can transfer HCV-RNA to trigger IFN production in neighboring pDCs	_	Dreux et al. (2012)
	HLSECs	_	HCV-infected hepatocytes	Exosomes from supernatants of HLSECs stimulated with IFNs can transfer antiviral products to infected hepatocytes	_	Giugliano et al. (2015)
	UMSCs	let-7f, miRNA-145, miRNA-199 and miRNA-221	Infected hepatocytes	Exosomal let-7f, miRNA-145, miRNA-199a, and miRNA-221 inhibits HCV replication by mediating RISC combined with viral RNA in infected cells	_	Qian et al. (2016a)

Santangelo et al. demonstrated that certain immunomodulatory miRNAs, including 122-5p, 222-3p, 146a, 150-5p, 30c, 378a- 3p, and 20a-5p, are enriched in exosomes derived from the sera of patients with chronic HCV infection. These exosomes can deregulate and reduce the expression of cytotoxicity-related mRNAs from intrahepatic NK cells to aid viral infection. The expression of these exosomal miRNAs (especially miRNA-122-5p or miRNA-222-3p) is markedly reduced following treatment with direct-acting antivirals (DAAs), which subsequently restores the functions of NK cells. Exosomal miRNAs can serve as biomarkers for monitoring the progression of liver disease and immune function (Santangelo et al., 2018). Notably, it has been reported that the activity of HCV in patients with rheumatoid arthritis (RA) who are suffering from chronic HCV infection is enhanced following rituximab therapy. The expression of exosomal miRNA-155 secreted from B cells has been found to be significantly higher in the sera of HCV-infected patients with RA compared to that of uninfected patients with RA. Rituximab can deplete B cells, and the expression of exosomal miRNA-155 was found to coincide with the number of B cells. The inhibitory effect of the alterations in the expression of B cell-derived exosomal miRNA-155 on HCV replication in hepatocytes suggested that rituximab may increase HCV-induced viremia in patients with RA by reducing the expression of exosomal miRNA-155 (Liao et al., 2018).

## Application of exosomes in clinical diagnoses

The biological features and functions of exosomes have been exploited for the diagnosis of HCV-related diseases and also for use as carriers of drug-targeting molecules. First, exosomal HCV RNA or miRNAs may serve as valuable biomarkers for evaluating therapeutic efficacy. Exosomes isolated from the sera of treatment-resistant patients have been reported to contain negative-sense HCV RNA, which was mostly associated with replication-competent HCV RNA, while exosomes isolated from the sera of non-responders lacked HCV RNA. The negative-sense HCV RNA in exosomes may be possibly implicated and measured for assessing treatment resistance with IFN/ribavirin (Bukong et al., 2014). Fan et al. observed that the expression of exosomal HCV RNA is significantly correlated with the expression of HCV RNA in the serum, and reported that the expression of exosomal HCV RNA was lowest in the group infected with genotype 1b (SVR) and highest in the group infected with genotype 6a (NR). Measuring the expression of exosomal HCV RNA may help to predict therapeutic efficacy (Fan et al., 2017). Exosomal miRNAs can also serve as biomarkers of therapeutic efficacy owing to their influence on treatment response. It has been reported that therapeutic efficacy varies highly across patients infected with different HCV genotypes. The majority of patients infected with genotype 1b were in the SVR group, whereas most patients infected with genotype 6a were in the NR group. The expression levels of exosomal miRNA-155 were similar to the loads of exosomal HCV RNA, which suggested that the expression of exosomal miRNA-155 was positively associated with HCV replication. Furthermore, the expression of exosomal miRNA-122 in the group infected with genotype 1b (SVR) was significantly higher than that of the groups infected with genotype 2a and genotype 6a, which indicated that the high exosomal expression of miRNA-122 could be associated with a positive therapeutic effect (Fan et al., 2017). Fan and coworkers also demonstrated that the expression of miRNA-122 in the sera and exosomes was higher in the SVR group than in the NR group, and that the expression of miRNA-199a was similar to that of miRNA-122 in the sera and exosomes. This indicated that miRNA-199a and miRNA-122 could serve as biomarkers for predicting therapeutic efficacy in patients infected with different genotypes of HCV (Jiao et al., 2017). It has been reported that the expression of certain immunomodulatory miRNAs is lower in exosomes isolated from the plasma of patients with chronic HCV infection following treatment with DAAs, and the reduction in the expression of exosomal miRNA-122-5p and miR-222-3p could serve as biomarkers of immune restoration (Santangelo et al., 2018). Furthermore, a previous study demonstrated that exosomal miRNA-16-5p obtained from saliva samples is strongly associated with the occurrence of primary liver cancer, and possibly serves as a valuable diagnostic marker (Petkevich et al., 2021).

Second, compared to pathologic examination, exosomes can serve as an early and non-invasive diagnostic biomarkers of HCV-related HCC. In 2020, Ghosh and coworkers observed that a combination of seven exosomal miRNAs, including 10b-5p, 221-3p, 223-3p, 10b-5p, 221-3p, 223-3p, and 21–5, serve as sensitivity markers for the early diagnosis of HCC regardless of HCV etiology, especially HCCs associated with low alpha-fetoprotein (Ghosh et al., 2020). A study reported that the expression of lncRNA-HEIH was highest in exosomes extracted from the sera of patients with HCV-related HCC, followed by that in exosomes derived from HCV-induced cirrhosis, and was lowest in exosomes derived from individuals with chronic HCV infection. LncRNA-HEIH in the serum and exosomes can be used to assess potential HCV-related HCC (Zhang et al., 2018). Matboli et al. reported that the expression of lncRNA-RP11-583F2.2 is higher in exosomes derived from the sera of patients with HCC compared to that of patients with HCV and healthy individuals. This finding revealed that exosomal lncRNA-RP11-583F2.2 can serve as a diagnostic and prognostic biomarker of HCC. However, it remains to be elucidated whether exosomal lncRNA-RP11-583F2.2 can be used as a biomarker of HCV-related HCC and regulator of HCV infection. In one study, the copy number of the circulating exosomal lnc-DANCR was used as a biomarker for predicting the recurrence and mortality of HCV-related HCC (Wang et al., 2021). In summary, the findings revealed that exosomes carrying RNAs or ncRNAs are emerging as biomarkers in viral research.

## Application of exosomes in clinical treatment

Exosomes have recently emerged as novel drug delivery tools for the treatment of various diseases. For instance, miR-122-transfected adipose tissue-derived mesenchymal stem cells (AMSCs) can deliver miRNA-122 to HCC cells *via* exosomes, which targeted to a disintegrin and metalloprotease 10 (ADAM10), insulin-like growth factor receptor (IGF1R) in HCC cells, and cyclinG1, thereby increasing the sensitivity of HCC cells to chemotherapeutic agents (Lou et al., 2015). Lou and coworkers also observed that exosomes originating from AMSCs with modified miRNA-122 transmitted miRNA-122 to HSCs. The transmitted miRNA-122 targeted certain genes, including the genes encoding IGF1R, cyclin G1, and prolyl-4-hydroxylase a1 in HSCs, which suppressed collagen maturation and proliferation of HSCs, thus protecting against liver fibrosis. These results suggested that exosomes can enhance the therapeutic effect of AMSCs that are resistant to liver fibrosis by transmitting modified miRNA-122 (Lou et al., 2017). Exosomes can also transfer small silencing RNAs targeting HCV or CD81 between hepatic cells *in vitro* and in mice, which extended the scope of RNA-interference (RNAi) -based therapeutics against HCV infections (Pan et al., 2012). Therapeutic peptides, some short sequences from natural peptides/proteins, are instability and vulnerability to enzyme degradation which confined their clinical application, although they have high biocompatibility, target specificity, therapeutic efficacy and low or non-toxicity *in vivo*. Fortunately, therapeutic peptides could be encapsulated in exosomes before being delivered to diseased organs (Bunggulawa et al., 2018). In addition to exosome-based drug delivery systems, other therapeutic approaches, including gene silencing and exosome-derived therapeutic approaches (such as tissue regeneration), have been developed. In summary, exosomes can be used for the diagnosis and treatment of liver diseases related to HCV infections, and could serve as next-generation nanomaterials. However, while exosomes have apparent advantages, some unresolved problems also exist. First, how loading exogenous drugs effectively into exosomes is a technical barriers to clinical application. Second, the methods and standards for assessing loading efficiency need to be urgently established for quality control of the exosomes. Additionally, exosomes can have possible unpredictable side effects as therapeutic tools.

## Discussion

Exosomes act as a “double-edged sword” in HCV infections. On one hand, they can promote viral infection by transferring several viral or cellular components to other uninfected cells and “mask” viral particles and RNA from antibody-mediated immune responses. On the other hand, exosomes containing antiviral substances or those derived from immune cells can inhibit viral infection. Exosomal cargo possibly serves for an efficient cellular adaptive mechanism for improving cellular survival under viral stress, or promotes the survival of virus itself during HCV infections.

Significant advances have been made with regards to understanding the role of exosomes in HCV infection. Nevertheless, further insights into HCV infections are necessary, especially for understanding host-virus interactions, intercellular communication, and immunomodulation. Modified exosomes can be used as biomarkers for assessing the severity of viral infections and therapeutic efficacy. Exosomes can also be used as biological, targetable, relatively stable, and promising vehicles for the delivery of therapeutic drugs against HCV infections and HCV-mediated liver diseases.

## Author contributions

YY prepared the first draft of the manuscript. YC, QC and YZ edited and revised the manuscript. LM designed and guided the review. All the authors have reviewed the final manuscript.

## Funding

This work was supported by the National Natural Science Foundation of China (grant number: 32070182) and the Key Project of Jiangsu Commission of Health (grant number: ZD2021049).

## Conflict of interest

The authors declare that the research was conducted in the absence of any commercial or financial relationships that could be construed as a potential conflict of interest.

## Publisher’s note

All claims expressed in this article are solely those of the authors and do not necessarily represent those of their affiliated organizations, or those of the publisher, the editors and the reviewers. Any product that may be evaluated in this article, or claim that may be made by its manufacturer, is not guaranteed or endorsed by the publisher.
